# Machine learning accelerates pharmacophore-based virtual screening of MAO inhibitors

**DOI:** 10.1038/s41598-024-58122-7

**Published:** 2024-04-08

**Authors:** Marcin Cieślak, Tomasz Danel, Olga Krzysztyńska-Kuleta, Justyna Kalinowska-Tłuścik

**Affiliations:** 1 Faculty of Chemistry, Jagiellonian University, Gronostajowa 2, 30-387 Kraków, Małopolska Poland; 2 Doctoral School of Exact and Natural Sciences, Jagiellonian University, Prof. S. Łojasiewicza 11, 30-348 Kraków, Małopolska Poland; 3 Computational Chemistry Department, Selvita, Bobrzynskiego 14, 30-348 Kraków, Małopolska Poland; 4 Faculty of Mathematics and Computer Science, Jagiellonian University, Prof. S. Łojasiewicza 6, 30-348 Kraków, Małopolska Poland; 5 Cell and Molecular Biology Department, Selvita, Bobrzynskiego 14, 30-348 Kraków, Małopolska Poland

**Keywords:** Machine learning, Virtual screening, Monoamine oxidase inhibitors, Molecular descriptors, Molecular docking, Cheminformatics, Medicinal chemistry

## Abstract

Nowadays, an efficient and robust virtual screening procedure is crucial in the drug discovery process, especially when performed on large and chemically diverse databases. Virtual screening methods, like molecular docking and classic QSAR models, are limited in their ability to handle vast numbers of compounds and to learn from scarce data, respectively. In this study, we introduce a universal methodology that uses a machine learning-based approach to predict docking scores without the need for time-consuming molecular docking procedures. The developed protocol yielded 1000 times faster binding energy predictions than classical docking-based screening. The proposed predictive model learns from docking results, allowing users to choose their preferred docking software without relying on insufficient and incoherent experimental activity data. The methodology described employs multiple types of molecular fingerprints and descriptors to construct an ensemble model that further reduces prediction errors and is capable of delivering highly precise docking score values for monoamine oxidase ligands, enabling faster identification of promising compounds. An extensive pharmacophore-constrained screening of the ZINC database resulted in a selection of 24 compounds that were synthesized and evaluated for their biological activity. A preliminary screen discovered weak inhibitors of MAO-A with a percentage efficiency index close to a known drug at the lowest tested concentration. The approach presented here can be successfully applied to other biological targets as target-specific knowledge is not incorporated at the screening phase.

## Introduction

Exploration of a large chemical space^1^ in the search for novel lead compounds remains a challenge^2^. Thus, modern drug discovery campaigns require fast, robust, and efficient approaches to accelerate the design process^3–5^. The recent remarkable development of computational methods and algorithms has led to the successful application of virtual screening (VS)^6^, often based upon molecular docking procedures. It is routinely applied to assess the affinity of a ligand to the selected target protein^7^. The structure-based techniques constantly evolve and improve due to the increasing number of data deposited within the Protein Data Bank (PDB)^8^. This database is the utmost source of structural information concerning intermolecular interactions in biological systems. Through a deeper understanding of protein-ligand complex formation and stabilization, novel algorithms can be introduced and subsequently modified. Thus, as a consequence, an advantageous route to increasing the predictive power of the methods applied may be obtained. The utility of molecular docking procedures in the continued search for new lead structures is often fraught with costly computations to discover the optimal binding pose for the screened compounds. Of late, such calculations are often complimented or entirely bypassed by machine learning (ML) methods, that can derive quantitative structure-activity relationship (QSAR) models based on the ligands’ chemical structures^9^. These models use different classes of molecular descriptors as input and return predicted activity, e.g. estimated binding affinity or IC$_{50}$ values. Nevertheless, the results of QSAR models are highly dependent on the training datasets, and predictions can be unreliable when novel chemotypes are presented to the model^10^.

In parallel to improving QSAR models, significant efforts are also being made to accelerate docking-based VS. Recently, there has been an exponential increase in available screening libraries, ranging from purchasable compounds through on-demand and combinatorial libraries to de novo generated chemical spaces. Using classical molecular docking procedures to screen billions of molecules is infeasible^2,11^. In consequence, the highly performing ML methods that predict docking scores based on two-dimensional molecular structures seem a good alternative^12^. A recent publication suggests that ML models can outperform single-conformation docking when trained with docking scores from protein conformation ensembles^13^. Finally, deep neural networks enable fast screening of over a billion compounds towards various molecular targets^14^. In this study, we employ ML methods to accelerate the discovery of new monoamine oxidase inhibitors (MAOIs) in constrained subspaces of VS libraries.

**Figure 1:**
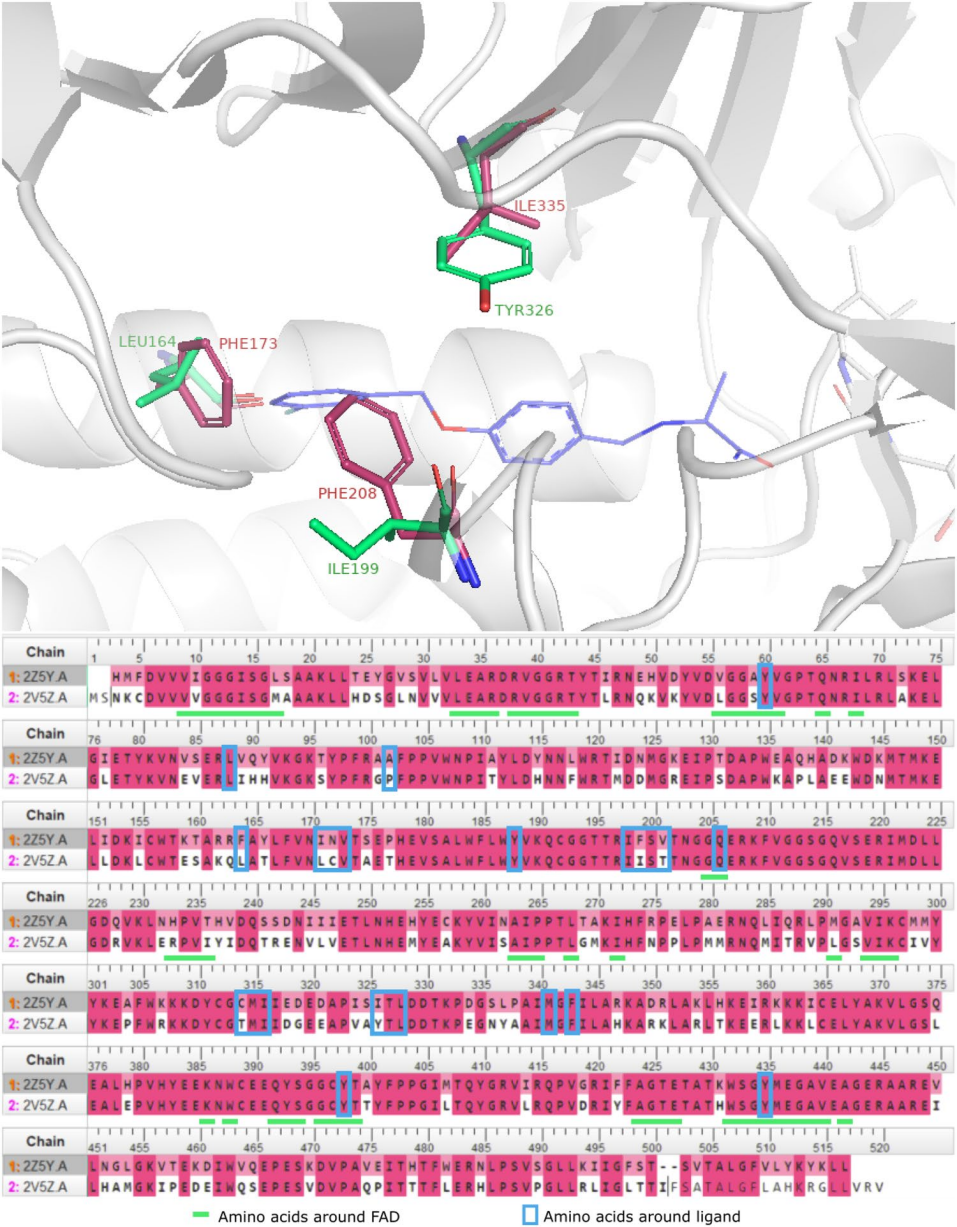
The superposition of MAO-A (2Z5Y) and MAO-B (2V5Z) binding sites (top). The differing amino acids are shown as red and green sticks for MAO-A and MAO-B, respectively. An exemplary ligand, ((S)-2[4-(3-fluorobenzyloxy)benzalamino]propanamide)in the MAO-B binding site, is shown in blue. In the MAO-A/MAO-B sequence alignment chart (bottom), the amino acids of the binding site are marked with a blue frame, and these near the FAD are underlined in green. The pocket comparison was created with PyMOL^15^, and the sequences were aligned with MOE^16^.

Presently, the number of patients suffering from central nervous system dysfunctions increases rapidly^17^. The complex and uncomprehended etiology causes that the discovery and development of new, safe, and efficient drugs against such pathological conditions remain elusive^18,19^. One of the intensively studied and promising targets are monoamine oxidase enzymes (two isoforms MAO-A and MAO-B)^20,21^ which are flavin-binding (FAD) proteases responsible for the oxidative deamination of diverse endo- and exogenous monoamines, e.g. neurotransmitters^22^. MAOs dysfunctions may lead to many disorders, including major depressive disorder, anxiety disorder, Parkinson’s, and Alzheimer’s disease^23–25^. Thus, the significance of MAO as a drug target in neurodegenerative disorders or even cancer treatment seems to be justified^26–28^.

Over the years, many small molecular inhibitors of monoamine oxidase (MAOIs) have been designed and developed. They can be classified into either non-selective or selective, and either reversible or irreversible inhibitors^27^. MAO-A inhibitors are used as antidepressants, and these which act on MAO-B slow down the progression of Parkinson’s or Alzheimer’s diseases^29,30^. The first generation of MAOIs was a class of irreversible non-selective antidepressants that were later withdrawn from the market due to the severe toxicity^31^ with multiple undesirable drug-drug and drug-food interactions^32,33^. For instance, MAO-B degrades tyramine contained in many foods, and the inhibition of this enzyme combined with the lack of dietary restrictions can lead to hypertension (so-called “cheese effect”) or even death^34,35^. Currently, MAOIs are not considered the first-choice drugs and are prescribed only in cases of treatment-resistant depression^36,37^. Thus, it became crucial to design novel, selective, and reversible monoamine oxidase inhibitors. Nevertheless, such a process remains a challenge, as both MAO isoforms share a high level of sequence identity. However, some small differences within the binding site may support the selective MAO-A or MAO-B inhibitors design. The sequence alignment (Fig. 1) reveals three crucial mutations within the ligand’s binding site (Phe208/Ile199, Phe173/Leu164 and Ile335/Tyr326, for MAO-A/MAO-B, respectively) that with the additional structural/cavity shape differences can be a road map leading to the discovery of selective inhibitors^27,38,39^.

Several computer-aided ligand- and structure-based drug discovery approaches have been employed in the search for novel and efficient MAO-A and/or MAO-B inhibitors^27,40,41^. Vilar et al.^42^ discussed the application of the 2D and 3D features to train ligand-based models, including multiple linear regression, partial least squares regression, linear discriminant analysis, comparative molecular field analysis (CoMFA), pharmacophore models, and neural networks. Lorenzo et al.^43^ evaluated caulerpin analogs in a ligand- and structure-based virtual screening to find potential inhibitory activity against MAO-B. Wang et al.^41^ employed hierarchical ligand-based methods to find selective MAOIs.

Despite the successful results of the aforementioned methods, designing new, selective, and reversible MAOIs is still a significant challenge for medicinal chemists. Thus, we developed a universal methodology based on the ensemble of machine learning models for the quick assessment of the compound activity, on the example of MAO inhibitors. In this approach, ligand-based QSAR models were trained to approximate the docking scores of the Smina docking software^44^. The results obtained were used to prioritize a large number of compounds retrieved from the ZINC database^45^, filtered by multiple models of pharmacophoric constraints. To test the performance of the proposed method, the top compounds were docked to MAO-A and MAO-B. The scoring function results obtained showed a strong correlation to the predictions from our model. Finally, the 24 top selected compounds were synthesized and in vitro tested, showing up to 33% MAO-A inhibition.

**Figure 2:**
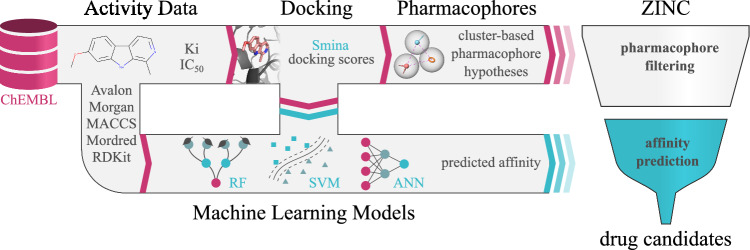
The schematic representation of the proposed virtual screening approach. The MAOs ligands selected from the ChEMBL database are docked, and the pharmacophore hypotheses of the best docking molecules are generated. In parallel, the fingerprints and descriptors of the docked compounds are applied to train machine learning models, allowing the prediction of activity values and docking scores. The pharmacophores and binding models are used to identify the most promising compounds from the ZINC database.

Unlike traditional QSAR models, the developed methodology is not limited by available bioactivity data and speeds up virtual screening compared to classical molecular docking procedures. In this study, the proposed approach is used to search for MAO-A and MAO-B inhibitors. Nevertheless, this methodology can be applied to other biological targets in general, allowing for the choice of molecular docking software which gives the best agreement to the experimental data. The methodology overview is depicted in Fig. 2.

## Materials and methods

### Activity dataset

**Figure 3:**
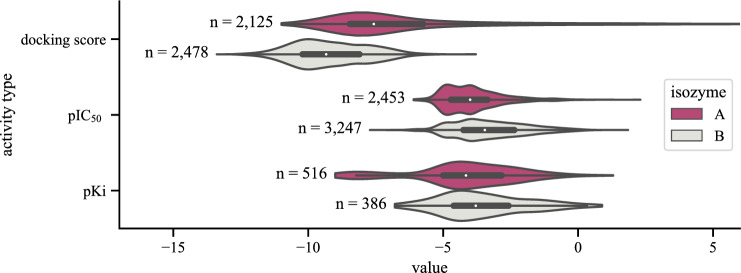
The distribution of the predicted (docking score) and experimental activity values retrieved from the ChEMBL database. The number of compounds is denoted by *n* and a color code was applied for each isozyme. The unit for docking scores is $\hbox {kcal}/\hbox {mol}$, and $-\hbox {log}_{10}\hbox {(nM)}$ for pIC$_{50}$ and pKi.

The MAO-A and MAO-B ligands with their corresponding activity data were downloaded from the ChEMBL database (ver. 29 2021-07-21)^46^. In the resulting dataset, there are 2 850 records with MAO-A and 3 496 records with MAO-B activity values. Only compounds with given Ki and IC$_{50}$ values were retained. Smina docking scores (DS) were calculated for the combined set of these compounds, filtered by molecular weight, excluding those greater than 700 Da, and highly flexible structures, for which docking procedure and precise pose predictions are more demanding and complicated. The distribution of the activity values used in the experiments and the docking scores obtained are shown in Figure 3. Due to the small number of available data, the compounds with given inhibition constants Ki were not used for activity modeling by machine learning methods. The IC$_{50}$ values were transformed into pIC$_{50}$ values ($\text {pIC}_{50}=-\log _{10} \text {IC}_{50}$) to mitigate the negative impact of very high values.

#### Data-splitting strategies

In the machine learning experiments, the prediction of two parameters was under investigation, these were pIC$_{50}$ values and docking scores. To train machine-learning models, the dataset was randomly split into training, validation, and testing subsets in the proportions of 70/15/15. The splitting was repeated five times to account for the variability of the data, and the mean score with its standard deviation was reported in all of the following results. In other experiments, the data was divided into subsets based on compound Bemis-Murcko scaffolds^47^. The proportions were kept the same as for the random split, and the overlap of the scaffolds between subsets was minimized to ensure that the evaluations were performed on chemotypes that differed from those used in the training process. This method of data splitting is used to test the model’s ability to generalize to new chemotypes. The scores achieved by the models for this data-splitting strategy are usually lower, but they describe the screening capability of these models more accurately.

To avoid splits with big differences in the distribution of the activity measurements, we sampled 50 splits and retained those with the lowest *D* statistic in the two-sample Kolmogorov–Smirnov (KS) test comparing the distribution of the activity labels in the training, validation, and testing subsets. The details of our KS data split are included in the Supporting Information.

### Molecular docking

Human monoamine oxidase (hMAO) crystal structure coordinates were downloaded from the Protein Data Bank (PDB)^8^. The resolution of the diffraction data for the selected structures of MAO-A with harmine (PDB ID: 2Z5Y)^48^ and MAO-B with safinamide (PDB ID: 2V5Z)^49^ was reported as 2.17 Åand 1.60 Å, respectively. Prior to the docking procedures, the ligands and water molecules were removed, so the only remaining molecules were the target enzyme and FAD. The active sites of both MAO isoforms are compared in Fig. 1.

The Smina docking software version 2020.12.10^44^ (https://sourceforge.net/projects/smina/) was used to perform molecular docking. This program is based on Autodock Vina^50^ and focuses on improving scoring and minimization. The initial 3D conformations of ligands were computed using the OpenBabel tool^51^. The docking procedure was run with the default parameters.

For comparison, other docking programs were used, such as AutoDock implemented in Yasara^52^, MOE^16^, and DockThor^53^. These programs were selected to compare a variety of both the conformation search algorithms and the scoring functions applied. To search the conformational space, AutoDock and DockThor use the Lamarckian and DMRTS (Dynamic Modified Restricted Tournament Selection)^54^ genetic algorithms, respectively, while Smina uses the ILS (Iterated Local Search) optimizer combined with the BFGS (Broyden–Fletcher–Goldfarb–Shanno) algorithm for local optimization. An empirical free-energy function is used for scoring in AutoDock and Smina, and DockThor uses a physics-based scoring function derived from the MMFF94S (Merck Molecular Force Field)^55^. MOE uses the Triangle Matcher algorithm for selecting conformations and scores them using the London dG scoring function.

### Activity prediction with machine learning models

#### Molecular descriptors

As input to machine learning models, several molecular descriptors and fingerprints were selected and applied. Molecular descriptors were calculated using Mordred^56^ and RDKit toolkits^57^, yielding 1 314 and 196 properties, respectively. These descriptors encode information about, e.g., the occurrence of individual fragments in molecules (characteristic functional groups), graph topological indexes, molecular weight, polar surface area, and other molecular properties. Some of them require initial information about the three-dimensional structure, e.g. Mordred which assigns 1D, 2D, and 3D descriptors. For optimizing molecular conformations, the MMFF^55^ implemented in the RDKit tool was used.

In the category of fingerprints, MACCS (Molecular ACCess System) keys^58^, Morgan^59^, and Avalon^60^ fingerprints were selected. The first type of fingerprint is based on a handcrafted set of predefined substructures. The Morgan fingerprint is a circular fingerprint (we use a radius of 2 and a vector length equal to 1024), and the Avalon fingerprint is path-based (we use 512 bits). The RDKit implementation of these fingerprints was applied.

#### Machine learning models

In the experiments, three machine learning algorithms widely used for molecular property prediction were employed: random forest (RF)^61^, support vector machine (SVM)^62^, and artificial neural network (ANN)^63^.

RF is a nonlinear model that builds multiple decision trees that create predictions by making consecutive binary decisions up to the point where the input data is sorted into a group with an assigned prediction value. The final prediction is retrieved from the predictions of all decision trees. RFs can process high-dimensional data such as molecular fingerprints effectively. They are interpretable, and their predictions can be attributed to the input features. On the other hand, a significant amount of time may be needed to train RFs on large datasets.

SVM is a model that constructs a regression formula optimized so that the majority of true values lie within an $\varepsilon$-margin from the predicted value. The nonlinearity of this model is achieved by applying the so-called kernel trick. SVMs are flexible and can process large datasets, but they are not interpretable and their computational complexity increases rapidly with the number of input features.

ANN is a biologically inspired model based on the way the neural network processes information. The model consists of many connected processing units called neurons. Each neuron can take as an input multiple features which are weighted by the learned strengths of neural connections. Neurons aggregate this information with the sum operation, use a non-linear activation function, and propagate the information to the next layer of neurons. The model prediction is the output of the network’s last-layer neurons. ANNs can handle big datasets and process large numbers of input features. They require almost no feature engineering because their initial layers can serve as data preprocessors. Unfortunately, these models are not interpretable and their performance depends heavily on the selection of the network architecture and training procedure.

#### Model evaluation

Multiple models were trained with different hyperparameters on the training set and then evaluated on the validation set to find the optimal hyperparameter set. Next, models were evaluated on the testing set, and test performance was reported for each combination of molecular descriptors and machine-learning models. The full set of tuned hyperparameters is included in Supporting Information.

The coefficient of determination R$^2$ was used for model evaluation. This evaluation metric describes how much variation of the true activity value is explained by the model, where the maximum possible value is 1 means that the model predictions correlate perfectly with the true activity values. The metric is defined below.

1 $$\begin{aligned} R^2 = 1 - \frac{\sum _{i=1}^N(y_i-\hat{y}_i)^2}{\sum _{i=1}^N(y_i - \bar{y})^2}, \end{aligned}$$

where *N* is the size of the testing set, $y_i$ is the true activity value of the *i*-th compound, $\hat{y}_i$ is the predicted activity value of the *i*-th compound, and $\bar{y}$ is the mean activity value in the testing set.

### Biochemical assay

The HTS screening was performed using the fluorometric assay: Monoamine Oxidase-A Inhibitor Screening Kit (Merck) according to the manufacturer’s protocol. Echo 650 Liquid Handler (Labcyte) was used to dose compounds on the 384-well plate format at 3 different concentrations: 100 $\upmu \hbox {M}$, 10 $\upmu \hbox {M}$, and 1 $\upmu \hbox {M}$ in duplicate. All compounds were dissolved in DMSO (at a final concentration 1%). Using Mantis Liquid Dispenser (Formulatrix), to each tested compound 12.5 $\upmu \hbox {L}$ of protein was added (at final concentration 56 nM) and incubated for 60 min at 25 $^{\circ }\hbox {C}$. After that, the enzymatic reaction was initiated by the addition of 10 $\upmu \hbox {L/well}$ of an aqueous solution of p-tyramine (substrate) and incubated for 60 min at 25$^{\circ }\hbox {C}$. The fluorescence intensity was measured on a plate reader (BioTek Synergy H1) using the following settings: excitation at 535 nm and emission at 587 nm. The data were normalized to low control (assay buffer containing substrate) and high control (protein and substate). The results were presented as a percentage of inhibition.

## Results

In this section, we explain the decisions made to optimize the VS pipeline (cf. Fig. 2) and the steps undertaken to select the best ligands that were chosen for the following in vitro tests. First, we discuss the reasons for the docking software choice. Second, the predictions of activity values and docking scores are compared between different machine learning methods and molecular descriptors or fingerprints. Next, the best models are ensembled (combined) to further improve prediction accuracy. Finally, the selected ensemble models are applied to search a pharmacophore-constrained chemical subspace, and the resulting diverse hits are confirmed in vitro.

**Figure 4:**
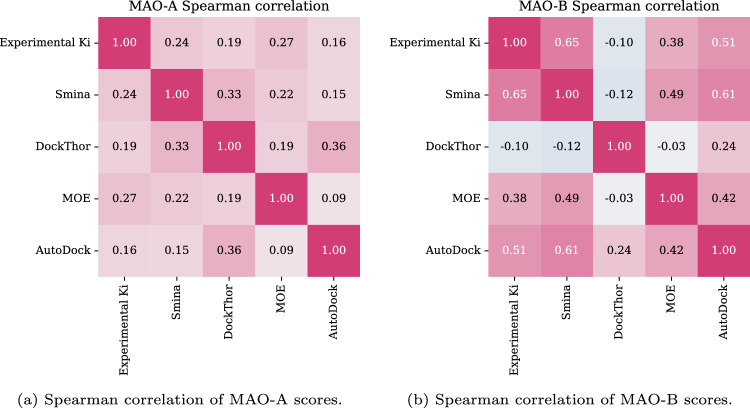
Correlation between selected scoring functions and experimental Ki for (**a**) MAO-A and (**b**) MAO-B isozymes.

### Selection of docking software and comparison of scoring functions performance

To select the docking software that shows the strongest correlation to the experimental activity data for both target systems, four available molecular docking tools were tested and compared. All the compounds deposited within the ChEMBL database with experimental Ki values for either MAO-A or MAO-B were docked (516 and 386 compounds, respectively). Subsequently, the correlation between docking scores and experimental Ki values was calculated and compared (Fig. 4). Due to the shift of experimental values in the MAO-B assays, the calculations for MAO-A and MAO-B were done differently. For MAO-A, we report the correlation of values assembled from all the assays. For MAO-B, we average correlation values computed separately for 5 assays with the greatest number of data points. More details on this approach are included in Supporting Information (see Figure C2).

The Spearman correlation coefficients suggest that all the docking programs achieve a rather weak correlation with the experimental Ki for MAO-A. In the case of MAO-B, Smina’s and Yasara’s (AutoDock) correlations are significantly higher. For further investigation, we decided to use Smina, considering its relatively good correlation with the experimental data for both molecular targets and the ease of use when building automated pipelines.

### Ligand-based activity prediction

The proposed VS pipeline starts with the activity data downloaded from the ChEMBL database. Multiple machine-learning models combined with different molecular representations/fingerprints were trained to predict the pIC$_{50}$ values of the compounds in the MAO-A and MAO-B assays. The calculated R$^2$-scores for two data splits of the activity dataset are presented in Table 1. We observe a moderate correlation between prediction and the experimental data for all models, reaching R$^2$=0.71 at the highest (random split). In the case of the scaffold split, the predictions performed for the testing subset are close to those obtained for the random split, with average R$^2$-scores dropping below 0 for the ANN that operates on the RDKit descriptors to predict MAO-B inhibition. The standard deviation of R$^2$-scores is also significantly higher for the scaffold split. However, this result is expected due to an insufficient number of data to learn/derive meaningful relationships that generalize to new chemical structures (there are only 1717 and 2272 compounds with IC$_{50}$ values in the MAO-A and MAO-B training sets, respectively). Additionally, one may observe that the highest scores are achieved for the Morgan and Avalon fingerprints, and even the MACCS fingerprint with a fixed set of hand-crafted structural features obtains competitive results. This suggests that the information about the chemical structure is crucial in predicting inhibitory activity, and the 1D descriptors (RDKit and Mordred) lack this information.

**Table 1:** Test R$^2$-scores in pIC$_{50}$ prediction for MAO-A and MAO-B inhibitors.

	MAO-A		MAO-B	
	Random	Scaffold	Random	Scaffold
RF				
Morgan	0.6121 ± 0.0384	0.3038 ± 0.1257	$\underline{0.6807 \pm 0.0296}$	$\underline{0.4444 \pm 0.0986}$
Avalon	0.6039 ± 0.0404	$\underline{{\textbf {0.3258}} \pm {\textbf {0.0971}}}$	0.6447 ± 0.0307	0.3724 ± 0.1310
MACCS	0.5888 ± 0.0408	0.2946 ± 0.1154	0.5862 ± 0.0444	0.3067 ± 0.1421
RDKit	0.5691 ± 0.0437	0.1778 ± 0.1400	0.6078 ± 0.0375	0.3816 ± 0.0795
Mordred	0.5279 ± 0.0169	0.1945 ± 0.1296	0.5916 ± 0.0336	0.4046 ± 0.0893
SVM				
Morgan	$\underline{{\textbf {0.6282}} \pm {\textbf {0.0309}}}$	0.2920 ± 0.1412	$\underline{{\textbf {0.7075}} \pm {\textbf {0.0277}}}$	$\underline{{\textbf {0.4923}} \pm {\textbf {0.0981}}}$
Avalon	0.6004 ± 0.0361	$\underline{0.3214 \pm 0.0745}$	0.6572 ± 0.0523	0.4115 ± 0.1203
MACCS	0.5757 ± 0.0203	0.2829 ± 0.1595	0.5717 ± 0.0482	0.3241 ± 0.1140
RDKit	0.5647 ± 0.0296	0.2443 ± 0.1407	0.6071 ± 0.0234	0.3982 ± 0.1561
Mordred	0.5855 ± 0.0418	0.2178 ± 0.1615	0.6567 ± 0.0478	0.3513 ± 0.3474
ANN				
Morgan	$\underline{0.6178 \pm 0.0540}$	0.2255 ± 0.1186	$\underline{0.6875 \pm 0.0314}$	0.4092 ± 0.1223
Avalon	0.5498 ± 0.0812	0.2453 ± 0.0494	0.6485 ± 0.0532	0.3728 ± 0.1721
MACCS	0.5841 ± 0.0500	$\underline{0.3025 \pm 0.1106}$	0.5745 ± 0.0418	0.3130 ± 0.1840
RDKit	0.5472 ± 0.0519	0.0947 ± 0.1805	0.6115 ± 0.0226	-0.1228 ± 1.1259
Mordred	0.5764 ± 0.0743	0.2085 ± 0.1590	0.6564 ± 0.0321	$\underline{0.4247 \pm 0.0819}$

The highest scores for each isozyme and split are typed in bold. Additionally, the highest scores for each isozyme, split, and model are underlined.

**Table 2:** Test R$^2$-scores in the prediction of Smina docking scores for MAO-A and MAO-B inhibitors.

	MAO-A		MAO-B	
	Random	Scaffold	Random	Scaffold
RF				
Morgan	0.7740 ± 0.0828	0.6066 ± 0.2859	0.6495 ± 0.0339	0.4143 ± 0.0536
Avalon	0.8218 ± 0.0668	0.5476 ± 0.4135	0.6648 ± 0.0490	0.3639 ± 0.1251
MACCS	0.7652 ± 0.0790	0.3996 ± 0.7031	0.6339 ± 0.0734	0.4649 ± 0.1259
RDKit	0.8788 ± 0.0447	0.8105 ± 0.0880	0.7228 ± 0.0638	0.5831 ± 0.1225
Mordred	0.8742 ± 0.0580	0.7924 ± 0.1034	0.7086 ± 0.0514	0.5906 ± 0.1023
SVM				
Morgan	0.8363 ± 0.0794	0.6019 ± 0.2581	0.7065 ± 0.0400	0.5020 ± 0.0880
Avalon	0.8513 ± 0.0657	0.5587 ± 0.3736	0.6752 ± 0.0522	0.3494 ± 0.1677
MACCS	0.7977 ± 0.0506	0.5251 ± 0.4731	0.6400 ± 0.0254	0.4798 ± 0.1452
RDKit	0.8888 ± 0.0464	0.8137 ± 0.1195	0.6902 ± 0.0508	0.6036 ± 0.1114
Mordred	0.8813 ± 0.0676	0.7765 ± 0.1701	$\underline{{\textbf {0.7248}} \pm {\textbf {0.0325}}}$	$\underline{{\textbf {0.6418}} \pm {\textbf {0.1076}}}$
ANN				
Morgan	0.8341 ± 0.0605	0.6380 ± 0.2676	0.6713 ± 0.0408	0.4106 ± 0.0353
Avalon	0.8357 ± 0.0349	0.6820 ± 0.2579	0.6742 ± 0.0596	0.4121 ± 0.0733
MACCS	0.8128 ± 0.0694	0.5023 ± 0.6289	0.6075 ± 0.0720	0.4273 ± 0.1485
RDKit	$\underline{{\textbf {0.8890}} \pm {\textbf {0.0335}}}$	$\underline{{\textbf {0.8243}} \pm {\textbf {0.0995}}}$	0.6829 ± 0.0495	0.5267 ± 0.1209
Mordred	0.8711 ± 0.0362	0.8227 ± 0.1028	$\underline{0.6952 \pm 0.0506}$	$\underline{0.6060 \pm 0.0871}$

The highest scores for each isozyme and split are typed in bold. Additionally, the highest scores for each isozyme, split, and model are underlined.

When working with experimental data, especially stored in public databases, numerous problems may arise from the differences in measurement methods (e.g., different assays), the precision of different devices used in the experiment, or even human errors. To overcome these discrepancies, the docking scores instead of the experimental data were used to train the same combinations of machine-learning models. For each compound in the activity dataset, molecular docking was performed to establish its Smina docking score, which was subsequently used for training. Table 2 demonstrates R$^2$-scores in the task of docking score prediction. In contrast to the prediction of pIC$_{50}$ values, the models obtained with this approach had considerably higher R$^2$-scores. The results for the scaffold split are still not satisfactory and exhibit higher variance but, in most cases, the gap between the random and scaffold split is not vast. Moreover, better scores are achieved using 1D descriptors, i.e., RDKit and Mordred. These results indicate that there is a strong (possibly nonlinear) correlation between selected molecular features and docking scores that is not observed in the biological data.

**Figure 5:**
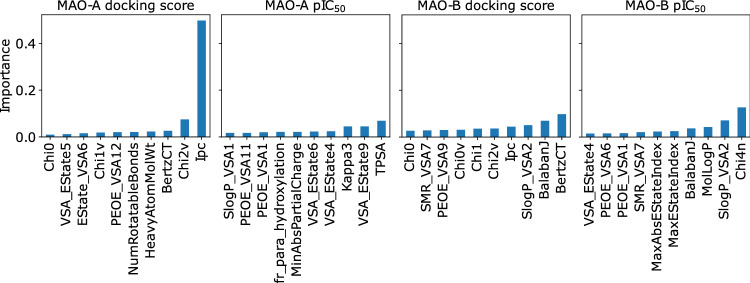
The feature importance in the prediction of docking scores and pIC$_{50}$ values for MAO-A and MAO-B.

#### Importance of input features

The deeper insight into the abovementioned observation revealed that different classes of molecular representations work best at predicting pIC$_{50}$ and docking scores, respectively. Interestingly, for the docking score prediction, the connectivity/shape/complexity molecular descriptors lead to better results, whereas for predicting the half-maximal inhibitory concentration, the substructural fingerprints representing molecular features perform better. The importance of the RDKit descriptors extracted from the random forest model on the docking score/pIC$_{50}$ prediction is shown in Fig. 5. These importance values correspond to the impurity decrease or, in other words, how much information is explained by the decisions that use these features.

The features important for predicting docking scores are dominated by topological descriptors (e.g. Ipc and BertzCT) constructed from the connectivity of molecular graphs and the number of heavy atoms or rotatable bonds. Conversely, the features selected when predicting pIC$_{50}$ values focus more on specific atom types and partial charges (e.g. TPSA and LogP), corresponding to interaction patterns in the protein-ligand complex. This finding confirms that docking scores correlate with simple molecular properties such as molecular weight and overall molecular shape. For reference, short explanations of the descriptors used in this analysis are presented in Table D2 in Supporting Information.

### Ensemble QSAR model

**Table 3:** The results of machine learning ensembles consisting of the 5 models with the best $R^2$ scores on the validation set.

		MAO-A		MAO-B	
		Random	Scaffold	Random	Scaffold
pIC$_{50}$	arithmetic	0.6531 ± 0.0421	0.3475 ± 0.0895	0.7212 ± 0.0276	0.4961 ± 0.0988
	$R^2$-weighted	0.6531 ± 0.0425	0.3477 ± 0.0884	0.7214 ± 0.0277	0.4977 ± 0.0987
DS	arithmetic	0.9044 ± 0.0452	0.7832 ± 0.2049	0.7525 ± 0.0428	0.6458 ± 0.0839
	$R^2$-weighted	0.9046 ± 0.0449	0.7833 ± 0.2048	0.7528 ± 0.0427	0.6462 ± 0.0839

**Figure 6:**
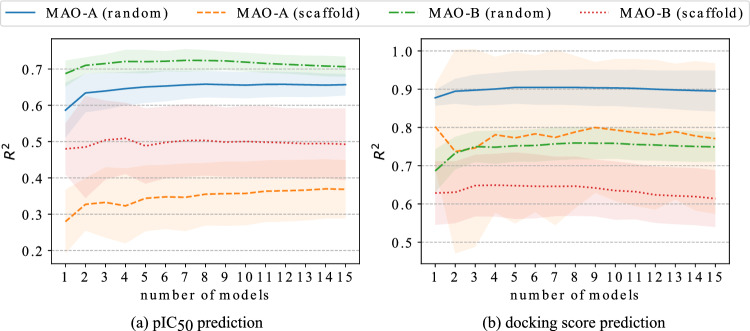
The relationship between the number of top models included in the ensemble and $R^2$ scores obtained for the testing set. The presented ensemble models use the $R^2$-weighted averages of predictions.

An important insight from the achieved results is that different models and descriptors can specialize in predicting different chemical structures. One may take advantage of this observation by combining multiple models and types of input data. We build an ensemble model consisting of several best-performing models by aggregating their predictions as follows:

2 $$\begin{aligned} \hat{y}(x; k) = \frac{\sum _{i=1}^k r^2_i\,\hat{y}_i(x) }{\sum _{i=1}^k r^2_i}, \end{aligned}$$

where *x* is the input compound and *k* is the number of best-performing models. We denote the prediction of *i*-th model by $\hat{y}_i(x)$ and its R$^2$-score calculated on the validation set by $r^2_i$. As the reasonable values of the R$^2$ metric are in the range [0, 1], the normalization of these values is not required, and they can be used directly as model weights so that predictions of more accurate models contribute stronger to the final prediction. The performance of this ensembling method (named “$R^2$-weighted” in Table 3) in comparison with the arithmetic mean of predicted pIC$_{50}$ and docking score (DS) values was evaluated. In this experiment, the top 5 models for each setup were chosen to create an averaged ensemble model. The difference in performance between weighted and non-weighted averages is negligible, so we conclude that both averaging strategies lead to similar gains. In the next step, the ensemble performance with various numbers of machine learning models was measured to select the number of models to be included in the ensemble. The results of this experiment are shown in Fig. 6. The obtained data suggest that using 5 models reasonably balances computation time and model performance.

### ML model performance in detecting active compounds

The performance of ML models in detecting active compounds was measured using the task of discerning active molecules from decoys. This method is often employed to assess docking results^64,65^. In this experiment, the strongest binders from ChEMBL are used as examples of active compounds, and decoys with a similar structure to the active compounds are generated. These decoys are designed to be inactive for the tested target. The performance of our ML models and a standard molecular docking protocol is compared using enrichment curves that describe what percentage of the active compounds is detected in the top X% of the molecules ranked by these models.

The three ML models with the highest R$^2$ scores for each isozyme were evaluated using the decoy recognition method described above. To conduct a reliable evaluation of the models, only molecules from the testing set were used in this experiment. Compounds with Ki less than 100 nM were selected and classified as actives. Decoys for these compounds were generated using the DUD-E server^66^. The testing sets consist of 7 actives versus 200 decoys and 28 actives versus 1200 decoys for MAO-A and MAO-B, respectively.

**Figure 7:**
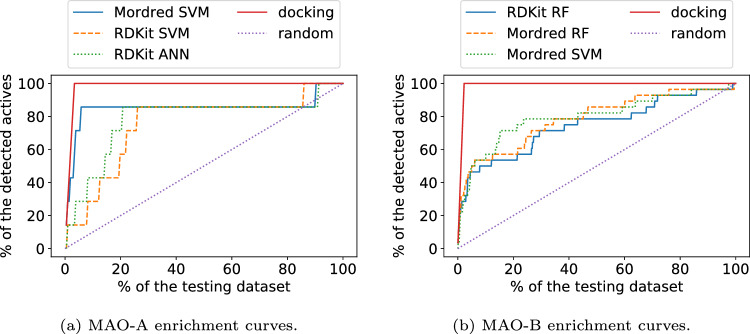
Enrichment curves calculated for Smina docking results and three best ML models on the testing set.

The ML model predictions and docking scores were used to rank all the compounds, and enrichment curves were plotted in Fig. 7 to show the ability of these models to detect active compounds in the top-ranked molecules. These results indicate that the tested models are capable of capturing a good portion of active compounds. We observe that by selecting only 10% of top molecules with respect to ML model predictions, we are able to capture $\sim$80% and $\sim$50% of true binders (known ligands) for MAO-A and MAO-B, respectively.

### Virtual screening with pharmacophoric constraints

A two-step VS procedure was conducted. In the first step, pharmacophore models for the best docking compounds from the activity data were defined. In the following step, the pharmacophore hypotheses were used to query the ZINC database^45^, and all the fetched compounds were evaluated using the developed ML activity models to select the most promising ligand candidates.

**Figure 8:**
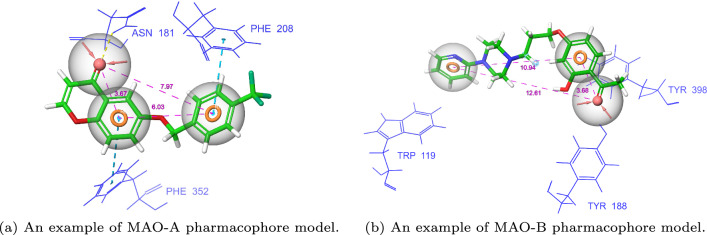
Examples of pharmacophore hypotheses generated based on the ChEMBL activity dataset and applied for putative ligands extraction from the ZINC database. (**a**) an exemplary compound (6-[[4-(trifluoromethyl)phenyl]methoxy]chromen-4-one) that conforms to one of the MAO-A pharmacophore hypotheses (**b**) an example of a compound (1-[2-hydroxy-4-[3-(4-pyridin-2-ylpiperazin-1-yl)propoxy]phenyl]ethanone) for one of the MAO-B pharmacophores.

#### Generation of diverse pharmacophore hypotheses

The *k*-means (*k*=50) clustering algorithm^67^ was used to extract groups of structurally similar compounds in the activity datasets described above. The algorithm used Morgan fingerprints as an input representation. Only the best compounds from each cluster were retained based on their docking scores. Next, these structurally diverse representatives were clustered using interaction fingerprints calculated by PLIP^68^, yielding 5 groups of compounds sharing similar ligand-protein interaction profiles. For each of the clusters, a pharmacophore hypothesis was postulated using PharmaGist^69^. Two exemplary pharmacophores are shown in Figure 8. All the other pharmacophore models are presented in SuppSupporting Information.

It is worth mentioning that the defined pharmacophore models were confronted against the MAO pharmacophores reported in the literature. In the case of MAO-A, our hypothesis is similar to the one proposed by Aljanabi et al.^28^ in which the active MAO-A compounds should contain two aromatic rings within the 6 Å distance. In our pharmacophore, the distance between the aromatic ring and hydrogen bond acceptor is defined as approx. 3.7 Å which was also suggested by Suryawanshi et al.^70^ Moreover, our proposed MAO-B pharmacophore hypotheses contain a motif of two aromatic rings together with a hydrogen bond donor. These hypotheses are supported by the literature that describes chalcones as a common motif in MAO-B inhibitors^71,72^.

#### Compound selection using pharmacophores and ML models

Subsequently, the ZINC database^45^ was searched for compounds that fulfill the pharmacophore requirements (7M for MAO-A and 5M for MAO-B). Then, all these molecules were evaluated using the developed ML activity models. For each compound, the mean prediction of the five best docking-score prediction models was calculated.

The compounds were clustered into structural groups using the *k*-means algorithm and the Tanimoto similarity index. The top molecules in six synthetically-accessible groups were selected for synthesis and biological testing. Sampling from different structural groups ensures the diversity of the selected compounds.

### Compound synthesis and MAO-A inhibition results

**Figure 9:**
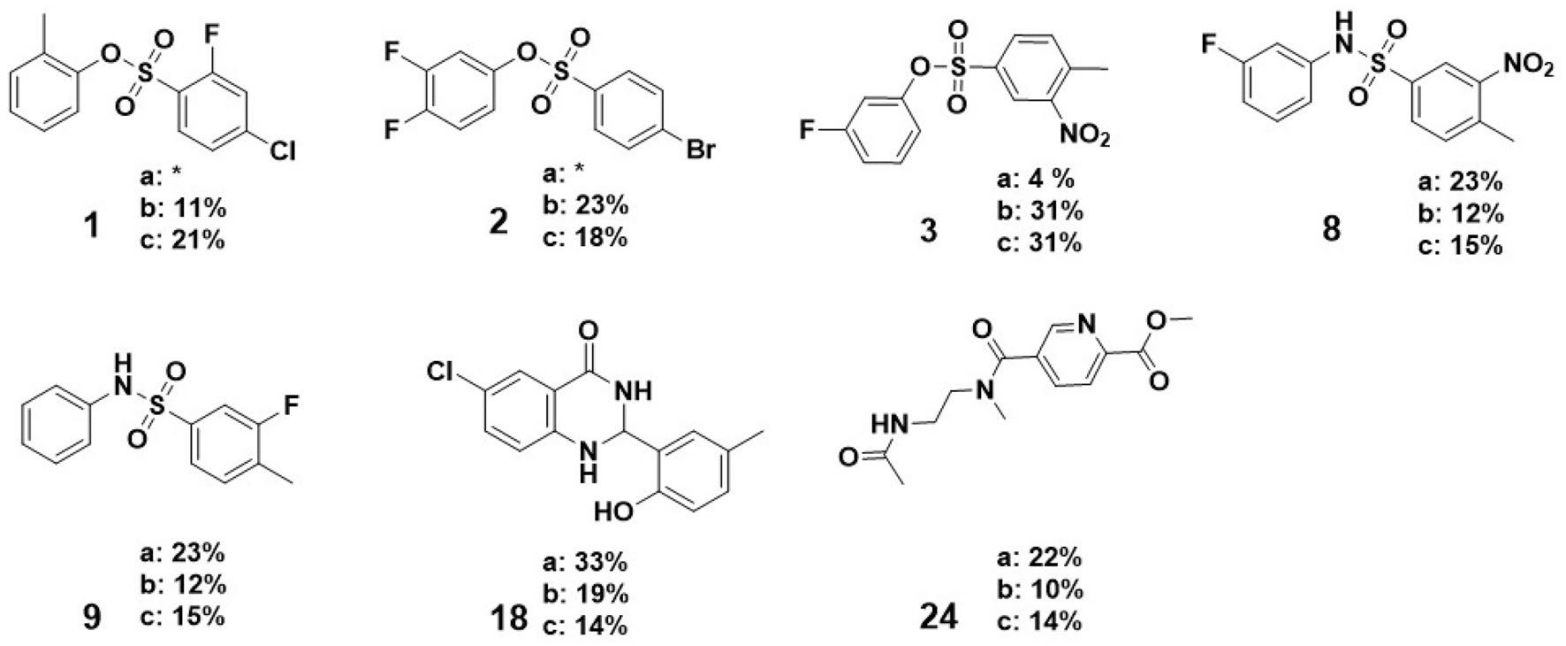
Selected compounds derived based on the presented ML protocol, showing the highest biological activity; (**a**–**c**) stands for the percentage of inhibition at 100, 10, and 1 $\upmu \hbox {M}$ concentrations of the tested compounds, respectively; * indicates either no inhibition or autofluorescence observed for the compound at the marked concentration level.

We selected four compounds from each of the identified six structurally diverse groups. These molecules were chosen based on their activity predictions, avoiding compounds with a high synthesis cost. In total, 24 compounds were selected, synthesized, and tested in the MAO-A biochemical assay. The synthesis protocols are described in Supporting Information. The compounds with the highest biological activity results are shown in Fig. 9.

The tested compounds achieved up to 33% MAO-A inhibition at the 100 $\upmu \hbox {M}$ concentration, and compound **3** obtained 31% inhibition at the 1 $\upmu \hbox {M}$ concentration. Importantly, the selected molecules are relatively small compared to the known MAO ligands, which makes them good starting candidates for further optimization. Nevertheless, we observed only moderate activity of the preliminarily selected compounds, which can be addressed by using more diverse screening libraries or training ML models on high-fidelity scoring functions based on molecular dynamics and quantum mechanics. The huge advantage of the presented screening methodology is the speed of hit identification from a large-scale database, enabling the first selection of candidates in about a week. Moreover, this approach can be easily modified and adapted to other targets and the best-performing docking procedures of choice.

The compounds synthesized and tested were relatively small with a molecular weight of around 300 Da. To properly compare our results with existing data, we decided to use the percentage efficiency index (PEI), which is a more suitable parameter for comparing compounds of different masses. PEI is calculated by dividing the percentage inhibition by the molecular weight in kDa.

#### The strongest inhibitor found in the MAO-A biochemical assay

At a concentration of 1 $\upmu \hbox {M}$, compound **3** achieved a PEI of 1.00, placing it 9th among 74 compounds in the ChEMBL database that were assigned inhibition percentages at the same concentration of 1 $\upmu \hbox {M}$. It is worth noting that the top-ranked compound on this list is a covalent inhibitor. Our compound comes close in terms of PEI to the known drug, moclobemide (PEI = 1.33), which is a monoamine oxidase inhibitor, indicating its potential as a new lead candidate.

**Figure 10:**
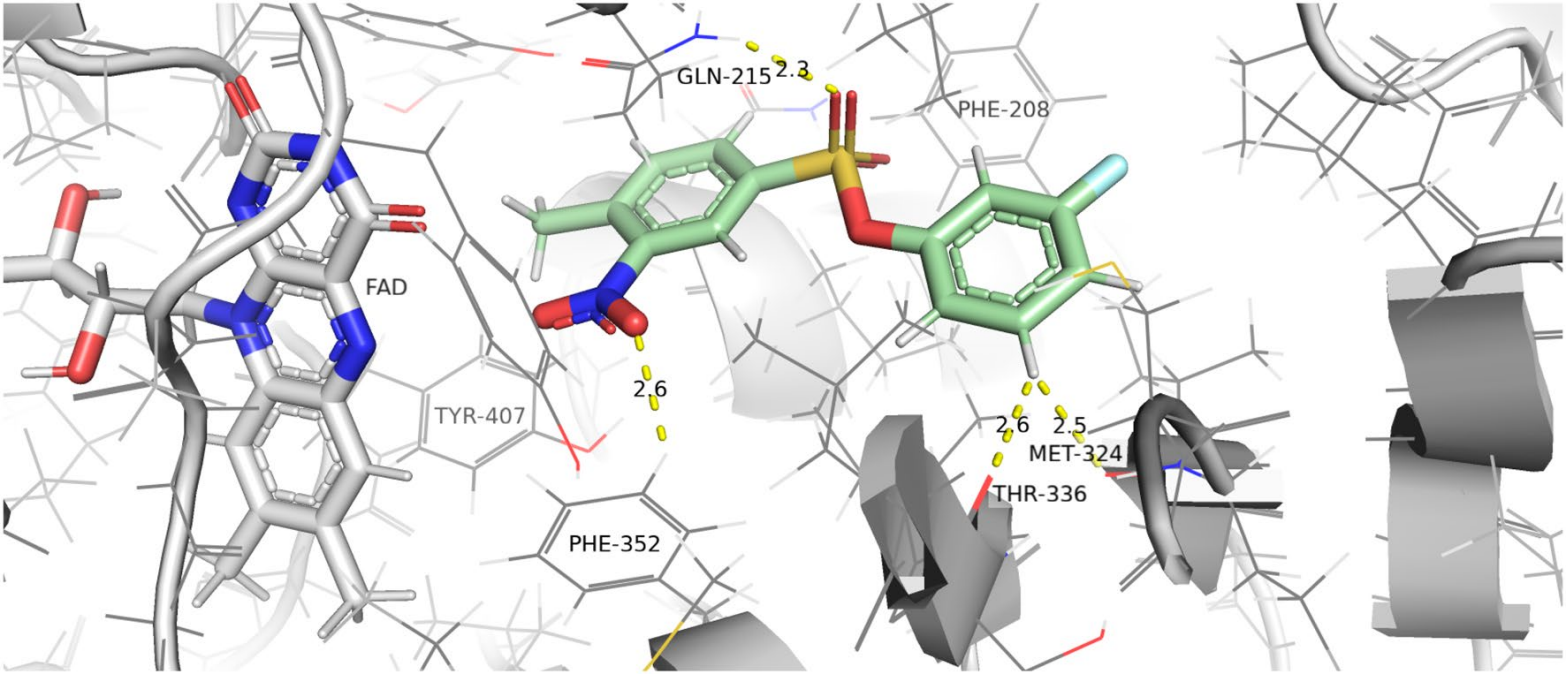
The proposed binding pose of the most active compound of all synthesized and tested in the MAO-A inhibition assay. The binding pose was visualized with PyMOL ^15^.

Molecular docking was conducted using the Smina package to propose a binding mode for this ligand. Three favorable poses were selected for molecular dynamics simulations of 30 ns to optimize and assess the obtained protein-ligand complex stability. The most promising pose, depicted in Figure 10, was found to be stable throughout the simulation time. Notably, during molecular dynamics, other less favorable ligand binding modes transform into a pose that is close to the proposed conformation.

In the predicted protein-ligand complex, a hydrogen bond interaction between the amine group of Gln215 and the sulfone oxygen of the ligand can be observed. Additionally, the stabilization of the sulfonyl group can be supported by the interaction of the Gln215 amide $\pi$ electrons and the aromatic ring of the ligand. The other aromatic ring of the small molecule interacts with the Met324 and Thr336 main chain oxygen atom of the peptide bond by C-H$\cdots$O contacts. Moreover, the $-\hbox {NO}_{2}$ group forms weak C-H$\cdots$O contact with Phe352 and $\pi$-$\pi$ with Tyr407 (classification based on the shortest observed distance between $\hbox {NO}_{2}$ and Tyr407). However, other studies suggest that the nitro group in the compounds inhibiting MAO forms cation-$\pi$ interactions with Tyr407^73^.

The proposed binding motif is consistent with similar examples in the literature postulating the nitro group of the compounds targeting MAO often orients itself towards the FAD cofactor^74^.

### VS acceleration achieved using the developed ML models

The advantage of using ML methods for docking score prediction instead of performing the traditional VS procedure by molecular docking is computation time reduction. To check this statement, the three random subsets of 1 000 molecules from the ZINC database were downloaded to perform VS using the Smina docking software and our best ML models. For MAO-A the best predictive models are 1st best: SVM on Mordred descriptors (random split), 2nd best: SVM on RDKit descriptors (random split), and 3rd best: SVM on Mordred descriptors (scaffold split). The top 3 models for MAO-B are 1st best: RF on RDKit descriptors (random split), 2nd best: RF on Mordred descriptors (random split), and 3rd best: RD on RDKit descriptors (random split). The last model in this comparison is the ensemble of the three best models that average their predictions.

**Table 4:** Comparison of the VS time using different methods.

VS method	MAO-A	MAO-B
Smina	14 900.0 s ± 330.5	19 160 s ± 2200.7
1st best	821.7 s ± 64.7	12.5 s ± 0.7
2nd best	11.8 s ± 0.1	844.0 s ± 55.4
3rd best	835.0 s ± 45.7	12.1 s ± 1.0
3-Ensemble	838.3 s ± 61.4	835.7 s ± 60.9

We compare the Smina docking procedure against the top 3 models for each isozyme. The 3-Ensemble model is the time of computing and averaging the predictions of the top 3 models.

In Table 4, we show the comparison of VS duration for the different approaches discussed above. We observe that all ML methods are more than an order of magnitude faster than the full docking procedure. Smina needs more than 4 hours to dock 1000 drug-like molecules, while even the ensemble model takes less than 15 minutes to score the same number of compounds. Moreover, the most time-consuming step in the developed ML methods is related to the computation of the molecular descriptors, and thus the time for models trained on Mordred descriptors increases compared to different approaches. When other features are used, e.g. RDKit descriptors, we can score 1000 molecules in less than 15 seconds.

All the computations were performed using an Intel Core i5 processor and 8 GB RAM. The standard deviation in Table 4 is reported for the 3 runs on different subsets of the ZINC database. Although the same computational resources were used to perform traditional and ML-based screening protocols, some ML methods, such as neural networks, can leverage GPUs to accelerate model training. Each model training run, including hyperparameter tuning, took less than a day. The NVIDIA GeForce GTX 1650 graphics card was used to train neural network models.

### Limitations

#### Applicability domain

Our approach can easily be adapted to other biological targets, and the code for training ML models is available online. However, a few constraints should be considered before employing our virtual screening package.

First, a high-resolution crystal structure of the protein target should be used to obtain docking scores of the compounds. These scores are then used to train ML models, so the results depend on the quality of the molecular docking protocol. Homology modeling or ML-based protein structure prediction tools, such as AlphaFold^75^ or ESMFold^76^, can be used to obtain protein structures for docking. However, the accuracy of these methods is often disputed.

The second consideration is the number of available ligands with activity measurements for the target. Active molecules are used to generate pharmacophore hypotheses and reduce the search space of druglike molecules. Moreover, activity data is used to train ML models. If insufficient data is provided, the screening results might be worse than those presented in this study.

#### Lack of high-fidelity methods

Our study is focused on reducing the time needed to propose the first set of compounds for a preliminary biochemical screen. Our virtual screening package can select a diverse pool of predicted binders in about a week. A considerable limitation of this study is the lack of high-fidelity methods used to confirm the potency of the selected compounds. Methods such as free-energy perturbation (FEP) or MM/GBSA are based on molecular dynamics and can produce predicted affinities that correlate better with the experimental results. We plan to explore the possibility of integrating these tools in the future. However, they can increase the virtual screening time significantly, which defies the main objective of this study.

The performance of the ML models can be also improved by using more consistent bioactivity data from one high-throughput screening campaign. Merging data from different sources may introduce significant noise^77^ and deteriorate the performance of QSAR models. Obtaining new activity measurements through biochemical assay delivers new high-fidelity compound binding data, but is more costly and time-consuming than most of the in silico methods.

## Conclusions

Nowadays, searching for new drug candidates in a constantly expanding chemical space remains a challenge for computational methods. However, developing new algorithms that incorporate both structure- and ligand-based methods, along with high-performance computing, can accelerate the drug discovery process. One promising strategy is the integration of machine learning techniques to increase the predictive power and level up the chance to conclude with a viable drug/lead candidate.

In this study, we demonstrated an approach where predictive ML-based models were used to derive docking scores instead of biological activity. We have shown that the model prediction does not significantly differ from the docking scores obtained in the classical molecular docking-based VS approach. Furthermore, the screening time using ML models is strongly decreased. The developed models return a docking score over 1000 times faster than the standard docking protocol. These models enable rapid screening of considerably larger compound libraries than docking-based approaches. Building QSAR models with this method is simple and allows for using unlabeled or generated data, rather than relying on external sources of often inconsistent biological assay results like those reported in the literature and assembled in the ChEMBL database. Our approach provides flexibility in choosing the docking program and scoring functions most aligned with the actual biological outcomes for the chosen target system.

The initial biological testing of compounds obtained using the proposed methodology to identify MAO-A inhibitors produced promising results. The 24 hit candidates were synthesized and tested, exhibiting up to 33% inhibition at the 1 $\upmu \hbox {M}$ concentration. Importantly, the PEI of the best selectee and a known drug moclobemide was comparable, which can be explained by the small size of our molecule relative to its inhibitory potency. This satisfactory initial outcome was achieved despite the small number of compounds that were selected for testing. We believe this general approach can prove successful in other screening projects.

### Supplementary Information

Supplementary Information.

## Data Availability

The data used for training QSAR models, including MAO-A and MAO-B activity data extracted from the ChEMBL database and computed docking scores, and the model training scripts are shared in our code repository: https://github.com/marcin-cieslak/mao-qsar.
