# Supplementary material for: Machine learning accelerates pharmacophore-based virtual screening of MAO inhibitors
Source: Sci Rep. 2024 Apr 8;14:8228. doi: 10.1038/s41598-024-58122-7 (PMC11369158; doi:10.1038/s41598-024-58122-7)
Supplement: Supplementary file 1 — Supplementary Information. [file 41598_2024_58122_MOESM1_ESM.pdf]

Supporting Information

Machine learning accelerates pharmacophore-based virtual screening of MAO inhibitors

Marcin Cieślak[1,2,3*], Tomasz Danel[4,1], Olga Krzysztyńska-Kuleta[5], Justyna Kalinowska-Tłuścik[1*]

[1]Faculty of Chemistry, Jagiellonian University, Gronostajowa 2, Kraków, 30-387, Małopolska, Poland.
[2]Doctoral School of Exact and Natural Sciences, Jagiellonian University, prof. S. Łojasiewicza 11, Kraków, 30-348, Małopolska, Poland.
[3]Computational Chemistry Department, Selvita, Bobrzynskiego 14, Kraków, 30-348, Małopolska, Poland.
[4]Faculty of Mathematics and Computer Science, Jagiellonian University, prof. S. Łojasiewicza 6, Kraków, 30-348, Małopolska, Poland.
[5]Cell and Molecular Biology Department, Selvita, Bobrzynskiego 14, Kraków, 30-348, Małopolska, Poland.

*Corresponding author(s). E-mail(s): marcin.cieslak@doctoral.uj.edu.pl; justyna.kalinowska-tluscik@uj.edu.pl;
Contributing authors: tomasz.danel@uj.edu.pl;

Appendix A Kolmogorov-Smirnov Data Split

The distribution of the activity values in public databases is often skewed and multimodal, and data splits (especially scaffold-based splits) can lead to huge differences in the distribution of labels in the training, validation, and testing subsets. To address this problem, we design a new splitting method for regression problems that ensures that both training and testing data cover the whole range of possible activity values. The splitting method is based on the two-sample Kolmogorov-Smirnov (KS) D statistic and can be combined with both random and scaffold-based data splits.

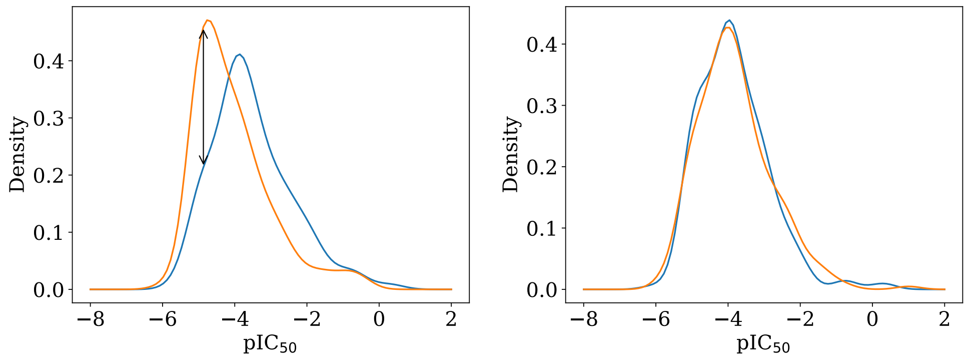

(a) Uncorrected scaffold-based split.

(b) KS scaffold-based split.

Fig. A1 The comparison between activity distribution in two subsets after performing a scaffold-based split; (a) an uncorrected scaffold-based split; the black arrow corresponds to the biggest difference between density functions; (b) a split selected from 10 generated scaffold-based splits using the described KS method.

The two-sample KS test is a nonparametric test used for comparing two one-dimensional distributions. The result is based on the D statistic that measures the maximum difference between the empirical distribution functions of two data samples, F_1 and F_2, using the following formula:

$$D = \sup_x |F_1(x) - F_2(x)|. \tag{A1}$$

In our data splitting approach, we generate more data splits using either a random or scaffold-based method and calculate the D statistic for each split taking the training and testing activity values as two data samples. Next, we keep only data splits with the lowest D statistic, ensuring that activity values in the training and testing subsets follow a similar data distribution. The result of this procedure is shown in Figure A1.

By using this KS approach to data splitting, we can train ML models to better capture the underlying distribution of activity values. However, a potential disadvantage of this method is that in scaffold-based splits some scaffolds may correspond to a separate mode in the activity distribution. These groups of compounds require to be counter-weighted by other compounds with similar activity, which may cause misinterpretation of the structure-activity relationship. We would like to flag this problem for future applications, even though we do not observe this issue for our data.

It is worth mentioning that, by employing the D statistic, only the maximum difference in the number of samples for one activity value is corrected. If the exact match of the distributions on the whole range of values is preferred, the Wasserstein distance may be applied instead. Another possible extension of the developed approach would be to implement an optimization method that swaps the data between training and testing subsets to minimize the D statistic instead of generating and filtering multiple data splits. We decide to use the filtering approach as it can be combined easily with random and scaffold-based splits.

Table B1 Set of ANN hyperparameters tested in a grid search.

hyperparameter	values
hidden size	1024, 512, 256
learning rate	0.0001, 0.001
number of layers	2, 3, 4, 5
dropout rate	0.2, 0.5
batch size	128, 256, 64

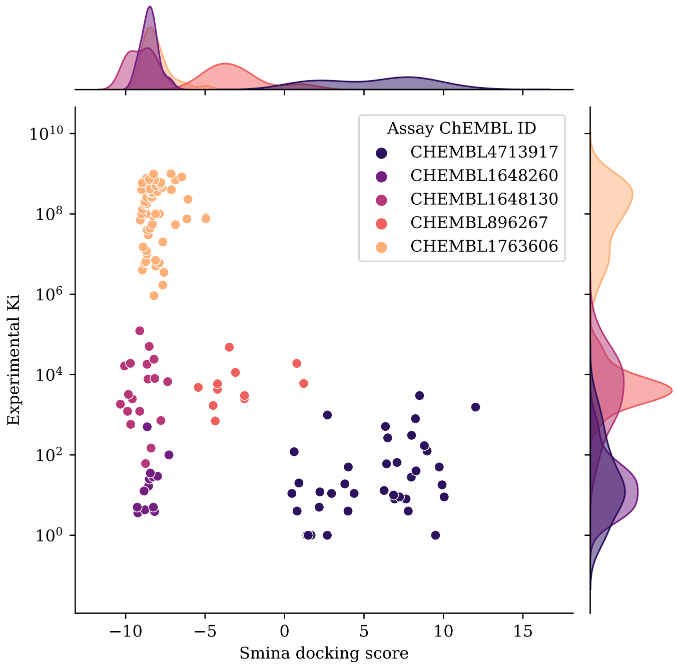

Fig. C2 The relationship between docking scores and experimental Ki presented for various assays retrieved from ChEMBL. The assay data is clearly separated in this visualization.

Appendix B Model Hyperparameters

A grid search was performed to select the best model for the prediction of activity values and docking scores. Each experiment was repeated for five different splits. The set of tested hyperparameters for the ANN model is provided in Table B1. ANNs were trained for 500 epochs with the Adam optimizer, using fully-connected layers followed by dropout and batch normalization layers. SVM was trained with the RBF kernel and a range of C values was tested $(2^0, 2^1, \ldots, 2^6)$. RF was run with the default set of parameters apart from the number of trees, which was set to up to 300 estimators.

Table D2 Short explanations of the descriptors used in the feature importance analysis.

descriptor	explanation
Chi0/1	Hall-Kier connectivity descriptor (2D) based on graph theory
Chi1/2v	Hall-Kier connectivity descriptor (2D) based on graph theory, including information about valence electrons for skeletal atom
Chi4n	Hall-Kier connectivity descriptor (2D) based on graph theory, including information about valence electron density (nVal) instead of valence
Kappa3	Hall-Kier topological descriptor (2D) which is a shape parameter determined by the extent of branching observed in the molecular graph.
BalabanJ	topological descriptor (2D), based on distance sums si as graph invariants; information about complexity/branching of the molecule
BertzCT	topological descriptor (2D), the general index of molecular complexity
Ipc	topological descriptor (2D); the information content of the coefficients of the characteristic polynomial of the adjacency matrix of a hydrogen-suppressed graph of a molecule
VSA_Estate	MOE-type descriptor (2D), using EState indices & van der Waals surface area
EState_VSA	MOE-type descriptor (2D) using EState indices & van der Waals surface area
PEOE_VSA	MOE-type descriptor (2D) including partial equalization of orbital electronegativities & van der Waals surface area
SlogP_VSA	MOE-type descriptor (2D) including logP & van der Waals surface area
SMR_VSA	MOE-type descriptor (2D) including molecular refractivity & van der Waals surface area
NumRotableBonds	constitutional descriptor (1D) giving the number of rotatable bonds
HeavyAtomMolWt	constitutional descriptor (1D) based on the average molecular weight of the molecule (excluding hydrogen atoms)
MinAbsPartialCharge	topological descriptor (2D); minimum absolute partial charge
MaxAbsEStateIndex	topological descriptor (2D); maximum absolute electrotopological index
TPSA	molecular property descriptor (2D); topological polar surface area
MolLogP	molecular property descriptor (2D); Wildman-Crippen logP
fr_para_hydroxylation	constitutional descriptor (1D); number of para-hydroxylation sites

Appendix C Binding Assay Miscalibration

We notice that the experimental data available in ChEMBL for MAO-A is inconsistent across different binding assays, as demonstrated in Figure C2. In this figure, we compare experimental Ki (log scale) and Smina docking scores for five assays containing the greatest number of compounds. The marginal distributions of the Ki values are significantly different for these assays.

What is more, the correlation between Ki and docking scores is positive for each of the assays. However, the overall correlation is negative when all data points are mixed. This result suggests that we cannot rely solely on the Ki measurements for this isoform, which further corroborates the usefulness of a standardized docking procedure as the binding approximation since learning the Ki values requires a correction of this data shift.

Appendix D Feature Importance Analysis

Short explanations of the features presented in the feature importance analysis are summarized in Table D2.

Appendix E Synthesized Compounds

Reactions were monitored by UPLC and TLC. Mass spectra: Shimadzu LCMS-2020 Single Quadropule Liquid Chromatograph Mass Spectrometer, Waters ACQUITY UPLC I-Class PLUS System. Products were purified by preparative TLC plates (silica gel G 500 µM 20×20 cm prep-scored) or flash column chromatography (PuriFlash Compact 420 or PuriFlash XS420, columns SIHP 50 µm). NMR spectra were recorded on Bruker Fourier 300 HD 300 MHz [^{1}H NMR (300 MHz)].

The synthesized compounds are shown in the figure E3.

E.1 Synthesis protocol for compounds (1-6)

To a stirred solution of proper acid chloride (50 mg, 1.0 eq) in DCM (1.5 mL, 30 vol) at 0 °C triethylamine (3.0 eq) and proper alcohol (1.0 eq) were added. The resulting mixture was stirred at 0 °C for 1 hour. Then the reaction mixture was warmed up to the room temperature, quenched with water and extracted with DCM. Organic phase was dried over anhydrous Na_2SO_4, filtered and concentrated. The crude product was purified by preparative TLC (eluting with hexane:ethyl acetate 4:1) to give desired product.

2-methylphenyl 4-chloro-2-fluorobenzene-1-sulfonate (1) 36 mg (Y=43%), UPLC purity: 99%.

^{1}H NMR (300 MHz, DMSO-d_6) δ 7.97 (dd, J = 10.2, 2.0 Hz, 1H), 7.86 (dd, J = 8.6, 7.7 Hz, 1H), 7.58 (ddd, J = 8.5, 2.0, 0.8 Hz, 1H), 7.39 – 7.31 (m, 1H), 7.30 – 7.16 (m, 2H), 7.00 – 6.92 (m, 1H), 2.17 (s, 3H).

3,4-difluorophenyl 4-bromobenzene-1-sulfonate (2) 69 mg (Y=51%), UPLC purity: 100%.

^{1}H NMR (300 MHz, DMSO-d_6) δ 7.94 – 7.87 (m, 2H), 7.84 – 7.76 (m, 2H), 7.51 (dt, J = 10.5, 9.1 Hz, 1H), 7.40 (ddd, J = 10.9, 6.8, 2.9 Hz, 1H), 6.97 – 6.87 (m, 1H).

3-fluorophenyl 4-methyl-3-nitrobenzene-1-sulfonate (3) 23 mg (Y=21%), UPLC purity: 100%, $[M\text{-}H]^-$=310.50 found.

^{1}H NMR (300 MHz, DMSO-d_6) δ 8.40 (d, J = 2.1 Hz, 1H), 8.09 (dd, J = 8.2, 2.1 Hz, 1H), 7.82 (d, J = 8.0 Hz, 1H), 7.53 – 7.41 (m, 1H), 7.30 – 7.21 (m, 1H), 7.18 – 7.11 (m, 1H), 7.03 – 6.93 (m, 1H), 2.63 (s, 3H).

methyl 4-[(3-fluoro-4-methylbenzenesulfonyl)oxy]benzoate (4) 29 mg (Y=34%), UPLC purity: 99%, $[M+H]^+$=325.00 found.

^{1}H NMR (300 MHz, DMSO-d_6) δ 8.03 – 7.92 (m, 2H), 7.77 – 7.70 (m, 1H), 7.65 – 7.56 (m, 2H), 7.28 – 7.18 (m, 2H), 3.85 (s, 3H), 2.35 (d, 1H).

3-fluorophenyl 4-chloro-2-fluorobenzene-1-sulfonate (5) 45 mg (Y=55%), UPLC purity: 99%.

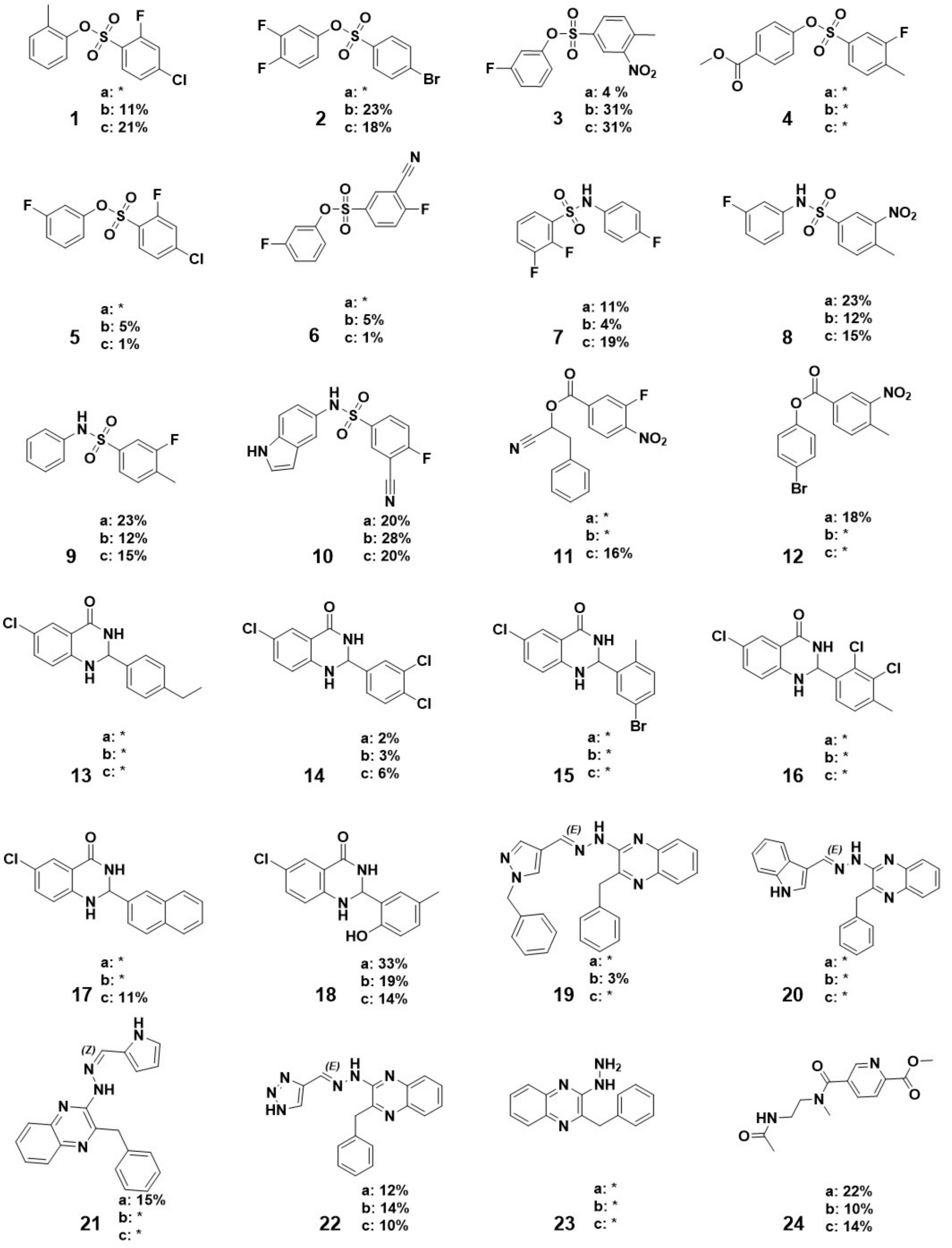

Fig. E3 Synthesized compounds and bioassay results. **a**, **b**, **c** is the percentage of inhibition at individual concentrations of the tested compounds 100, 10, and 1 µM, respectively. * indicates no inhibition or autofluorescence of the compound.

^{1}H NMR (300 MHz, DMSO-d_6) δ 7.96 (dd, J = 10.2, 2.0 Hz, 1H), 7.82 (dd, J = 8.6, 7.7 Hz, 1H), 7.59 – 7.51 (m, 1H), 7.50 – 7.42 (m, 1H), 7.31 – 7.21 (m, 1H), 7.15 (dt, J = 9.4, 2.4 Hz, 1H), 6.97 (dd, J = 8.3, 2.3 Hz, 1H).

3-fluorophenyl 3-cyano-4-fluorobenzene-1-sulfonate (6) 63 mg (Y=60%), UPLC purity: 99%, $[M\text{-}H]^-$=294.50 found.

^{1}H NMR (300 MHz, DMSO-d_6) δ 8.66 (dd, J = 5.8, 2.5 Hz, 1H), 8.29 – 8.20 (m, 1H), 7.81 (t, J = 9.0 Hz, 1H), 7.54 – 7.41 (m, 1H), 7.30 – 7.22 (m, 1H), 7.17 (dt, J = 9.5, 2.4 Hz, 1H), 6.98 (dd, J = 8.2, 2.3 Hz, 1H).

E.2 Synthesis protocol for compounds (7-10)

To a stirred solution of proper acid chloride (50 mg, 1.0 eq) in DCM (1.5 mL, 30 vol) at 0 °C pyridine (1.5 eq) and proper amine (1.0 eq) were added. The resulting mixture was stirred at 0 °C for 3 hours. Then the reaction mixture was warmed up to the room temperature and pyridine was removed by evaporation. Then the reaction mixture was quenched with water and extracted with DCM. Organic phase was dried over anhydrous Na_2SO_4, filtered and concentrated. The crude product was purified by preparative TLC (eluting with hexane : ethyl acetate 4:1) to give desired product.

2,3-difluoro-N-(4-fluorophenyl)benzene-1-sulfonamide (7) 53 mg (Y=41%), UPLC purity: 100%, $[M\text{-}H]^-$=286.50 found.

^{1}H NMR (300 MHz, DMSO-d_6) δ 10.73 (s, 1H), 7.75 (q, J = 8.2 Hz, 1H), 7.61 – 7.54 (m, 1H), 7.43 – 7.33 (m, 1H), 7.12 (d, J = 6.8 Hz, 4H).

N-(3-fluorophenyl)-4-methyl-3-nitrobenzene-1-sulfonamide (8) 27 mg (Y=24%), UPLC purity: 98%, $[M\text{-}H]^-$=309.60 found.

^{1}H NMR (300 MHz, DMSO-d_6) δ 10.79 (s, 1H), 8.33 (d, J = 2.0 Hz, 1H), 7.96 (dd, J = 8.1, 2.0 Hz, 1H), 7.70 (d, J = 8.1 Hz, 1H), 7.37 – 7.22 (m, 1H), 6.98 – 6.82 (m, 3H), 2.55 (s, 3H).

3-fluoro-4-methyl-N-phenylbenzene-1-sulfonamide (9) 25 mg (Y=49%), UPLC purity: 100%, $[M+H]^+$=266.70 found.

^{1}H NMR (300 MHz, DMSO-d_6) δ 10.29 (s, 1H), 7.47 – 7.41 (m, 3H), 7.28 – 7.17 (m, 2H), 7.12 – 6.98 (m, 3H), 2.25 (d, J = 2.2 Hz, 3H).

3-cyano-4-fluoro-N-(1H-indol-5-yl)benzene-1-sulfonamide (10) 24 mg (Y=24%), UPLC purity: 96%, $[M+H]^+$=316.00 found.

^{1}H NMR (300 MHz, DMSO-d_6) δ 11.10 (s, 1H), 10.00 (s, 1H), 8.17 (dd, J = 6.0, 2.4 Hz, 1H), 8.01 – 7.92 (m, 1H), 7.67 (t, J = 9.0 Hz, 1H), 7.32 (t, J = 2.8 Hz, 1H), 7.29 – 7.19 (m, 2H), 6.77 (dd, J = 8.6, 2.1 Hz, 1H), 6.37 – 6.32 (m, 1H).

E.3 Synthesis protocol for compounds (11-12)

To a stirred solution of proper acid chloride (50 mg, 1.0 eq) in DCM (1.5 mL, 30 vol) at 0 °C triethylamine (3.0 eq), proper alcohol (1.0 eq) and 4-dimethylaminopyridine (0.2 eq) were added. The resulting mixture was stirred at 0 °C for 1 hour and then overnight at room temperature. Reaction mixture was quenched with water and extracted with DCM. Organic phase was dried over anhydrous Na_2SO_4, filtered and concentrated. The crude product was purified by preparative TLC (eluting with hexane : ethyl acetate 4:1) to give desired product.

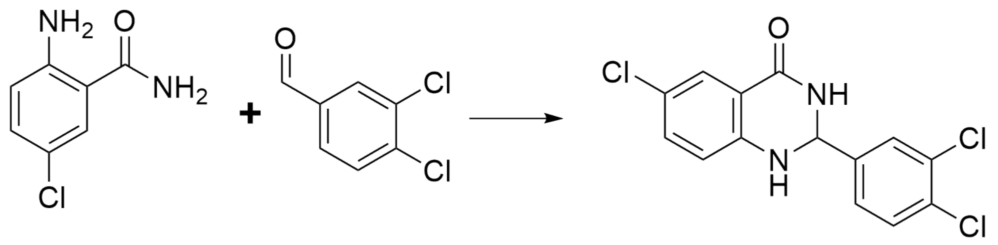

Fig. E4 Synthesis of compounds 13-18 illustrated by example of compound 14.

1-cyano-2-phenylethyl 3-fluoro-4-nitrobenzoate (11) 24 mg (Y=54%), UPLC purity: 95%, $[M\text{-}H]^-$=309.35 found.

^{1}H NMR (300 MHz, DMSO-d_6) δ 8.40 (d, J = 1.8 Hz, 1H), 8.12 (dd, J = 8.0, 1.9 Hz, 1H), 7.69 (d, J = 8.0 Hz, 1H), 7.41 – 7.35 (m, 2H), 7.34 – 7.16 (m, 3H), 5.77 – 5.70 (m, 1H), 3.22 (d, J = 6.7 Hz, 2H).

4-bromophenyl 4-methyl-3-nitrobenzoate (12) 23 mg (Y=56%), UPLC purity: 95%, $[M+H]^+$=339.10 found.

^{1}H NMR (300 MHz, DMSO-d_6) δ 8.60 (d, J = 1.8 Hz, 1H), 8.32 (dd, J = 8.0, 1.9 Hz, 1H), 7.76 (d, J = 8.1 Hz, 1H), 7.68 (d, J = 8.8 Hz, 2H), 7.40 – 7.27 (m, 2H), 2.64 (s, 3H)

E.4 Synthesis protocol for compounds (13-18)

The synthesis route for compounds 13-18 is presented in figure E4 and is based on the example of the synthesis of compound 14.

To a stirred solution of proper aldehyde (40 mg, 1.0 eq) in acetonitrile (1.5 mL, 30 vol), 2-Amino-5-chlorobenzamide (1.0 eq) and para-toluene sulfonic acid monohydrate (0.1 eq) were added. The resulting mixture was stirred at room temperature for 48 hours. Then the solvent was evaporated and the crude product was purified by preparative TLC (eluting with hexane:ethyl acetate 2:1) to give desired product.

6-chloro-2-[(3Z)-hepta-1,3-dien-2-yl]-1,2,3,4-tetrahydroquinazolin-4-one (13) 56 mg (Y=62%), UPLC purity: 95%, $[M+H]^+$=287.05 found.

^{1}H NMR (300 MHz, DMSO-d_6) δ 8.42 (s, 1H), 7.53 (d, J = 2.6 Hz, 1H), 7.38 (d, J = 8.1 Hz, 2H), 7.30 – 7.18 (m, 4H), 6.76 (d, J = 8.7 Hz, 1H), 5.74 (s, 1H), 2.59 (q, J = 7.6 Hz, 2H), 1.16 (t, J = 7.6 Hz, 3H).

6-chloro-2-(3,4-dichlorophenyl)-1,2,3,4-tetrahydroquinazolin-4-one (14) 7 mg (Y=7%), UPLC purity: 95%, $[M+H]^+$=328.60 found.

^{1}H NMR (300 MHz, DMSO-d_6) δ 8.59 (s, 1H), 7.74 – 7.63 (m, 2H), 7.53 (d, J = 2.6 Hz, 1H), 7.49 – 7.40 (m, 2H), 7.30 (dd, J = 8.7, 2.6 Hz, 1H), 6.79 (d, J = 8.7 Hz, 1H), 5.85 – 5.81 (m, 1H).

2-(5-bromo-2-methylphenyl)-6-chloro-1,2,3,4-tetrahydroquinazolin-4-one (15) 23 mg (Y=25%), UPLC purity: 96%, $[M+H]^+$=352.90 found.

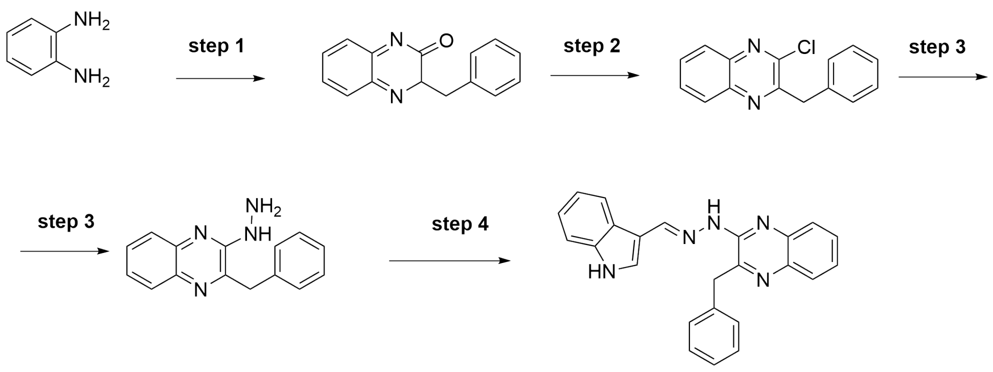

Fig. E5 Synthetic route for compounds 19-23.

^{1}H NMR (300 MHz, DMSO-d_6) δ 8.31 (s, 1H), 7.66 (d, J = 2.2 Hz, 1H), 7.58 (d, J = 2.6 Hz, 1H), 7.48 (dd, J = 8.1, 2.2 Hz, 1H), 7.31 (dd, J = 8.7, 2.6 Hz, 1H), 7.21 (d, J = 8.2 Hz, 1H), 7.13 (s, 1H), 6.78 (d, J = 8.7 Hz, 1H), 6.02 (s, 1H), 2.37 (s, 3H).

6-chloro-2-(2,3-dichloro-4-methylphenyl)-1,2,3,4-tetrahydroquinazolin-4-one (16) 72 mg (Y=76%), UPLC purity: 95%, $[M+H]^+$=341.00 found.

^{1}H NMR (300 MHz, DMSO-d_6) δ 8.42 (s, 1H), 7.57 (d, J = 2.6 Hz, 1H), 7.47 (d, J = 8.0 Hz, 1H), 7.40 (d, J = 8.0 Hz, 1H), 7.33 – 7.24 (m, 2H), 6.78 (d, J = 8.7 Hz, 1H), 6.15 (t, J = 1.9 Hz, 1H), 2.39 (s, 3H).

6-chloro-2-(naphthalen-2-yl)-1,2,3,4-tetrahydroquinazolin-4-one (17) 61 mg (Y=59%), UPLC purity: 97%, $[M+H]^+$=309.00 found.

^{1}H NMR (300 MHz, DMSO-d_6) δ 8.56 (s, 1H), 7.99 – 7.87 (m, 4H), 7.67 (dd, J = 8.4, 1.8 Hz, 1H), 7.61 – 7.50 (m, 3H), 7.41 (s, 1H), 7.29 (dd, J = 8.7, 2.6 Hz, 1H), 6.79 (d, J = 8.7 Hz, 1H), 5.97 (s, 1H).

6-chloro-2-(2-hydroxy-5-methylphenyl)-1,2,3,4-tetrahydroquinazolin-4-one (18) 15 mg (Y=13%), UPLC purity: 94%, $[M+H]^+$=389.10 found.

^{1}H NMR (300 MHz, DMSO-d_6) δ 9.62 (s, 1H), 8.08 (s, 1H), 7.54 (d, J = 2.6 Hz, 1H), 7.25 (dd, J = 8.7, 2.6 Hz, 1H), 7.14 (d, J = 2.2 Hz, 1H), 7.01 – 6.89 (m, 2H), 6.77 (dd, J = 10.5, 8.4 Hz, 2H), 5.99 (s, 1H), 2.17 (s, 3H).

E.5 Synthesis protocol for compounds (19-23)

The synthesis route for compounds 19-23 is shown in figure E5.

Step 1

To a solution of benzene-1,2-diamine (1.5 g, 1.0 eq) in ethanol (45 mL, 30 vol), phenylpyruvic acid (1.0 eq) was added. The reaction mixture was stirred at room temperature for 3h at microwave reactor. After reaction completion, solvent was evaporated and the crude product was purified by flash column chromatography (eluting

with gradient DCM:[DCM:MeOH = 9:1] 1:0 → 1:1) to afford 798 mg (Y=25%) of 3-benzyl-1,2-dihydroquinoxalin-2-one with 75% UPLC purity. UPLC (ESI): exact mass for $C_{15}H_{12}N_2O$: 236.09; $[M+H]^+$=237.30 found.

Step 2

To a stirred solution of 3-benzyl-1,2-dihydroquinoxalin-2-one (798 mg, 1.0 eq) in THF (24 mL, 30 vol) at room temperature, triethylamine (3.0 eq) and triphosgene (3.0 eq) were added. The resulting mixture was stirred overnight at 60 °C. Then the reaction mixture was quenched with water and extracted with DCM. Organic phase was dried over anhydrous Na_2SO_4, filtered and concentrated. The crude product was purified by flash column chromatography (eluting with gradient hexane : ethyl acetate 1:0 → 1:1) to afford 328 mg (Y=38%) of 2-benzyl-3-chloroquinoxaline with 80% UPLC purity. UPLC (ESI): exact mass for $C_{15}H_{11}ClN_2$: 254.06; $[M+H]^+$=255.10 found.

Step 3

To a stirred solution of 2-benzyl-3-chloroquinoxaline (328 mg, 1.0 eq) in ethanol (10 mL, 30 vol) at room temperature, hydrazine monohydrate (10.0 eq) was added. The resulting mixture was stirred overnight at 60 °C. Then the reaction mixture was quenched with water and extracted with DCM. Organic phase was dried over anhydrous Na2SO4, filtered and concentrated. The crude product was purified by flash column chromatography (eluting with gradient DCM:[DCM:MeOH = 9:1] 1:0 → 1:1) to afford 137 mg (Y=43%) of 2-benzyl-3-hydrazinylquinoxaline with 85% UPLC purity. UPLC (ESI): exact mass for $C_{15}H_{14}N_4$: 250.12; $[M+H]^+$=251.55 found.

Step 4

To a stirred solution of 2-benzyl-3-hydrazinylquinoxaline (25 mg, 1.0 eq) in methanol (1 mL, 30 vol) at room temperature, proper aldehyde (1.0 eq) was added. The resulting mixture was stirred overnight at room temperature. After reaction completion, solvent was evaporated and the crude product was purified by preparative TLC (eluting with DCM : acetone 9:1) to give desired product.

2-benzyl-3-[(2E)-2-[(1-benzyl-1H-pyrazol-4-yl)methylidene]hydrazin-1-yl]quinoxaline (19) 17 mg (Y=38%), UPLC purity: 93%, $[M+H]^+$=419.20 found.

^{1}H NMR (300 MHz, DMSO-d_6) δ 10.72 (s, 1H), 8.50 – 8.37 (m, 1H), 8.28 (s, 1H), 8.11 – 7.95 (m, 1H), 7.80 (s, 1H), 7.58 – 7.45 (m, 2H), 7.45 – 7.25 (m, 8H), 7.25 – 7.17 (m, 2H), 7.14 – 7.04 (m, 1H), 5.37 (d, J = 15.0 Hz, 2H), 4.27 – 4.08 (m, 2H).

2-benzyl-3-[(2E)-2-[(1H-indol-3-yl)methylidene]hydrazin-1-yl]quinoxaline (20) 3 mg (Y=9%), UPLC purity: 93%, $[M+H]^+$=378.20 found.

^{1}H NMR (300 MHz, DMSO-d_6) δ 11.71 (s, 1H), 10.54 – 10.33 (m, 1H), 8.77 (s, 1H), 8.51 – 8.44 (m, 1H), 7.99 (d, J = 2.7 Hz, 1H), 7.56 – 7.42 (m, 4H), 7.39 – 7.25 (m, 4H), 7.27 – 7.13 (m, 3H), 7.06 (t, 1H), 4.17 (s, 2H).

2-benzyl-3-[(2Z)-2-[(1H-pyrrol-2-yl)methylidene]hydrazin-1-yl]quinoxaline (21) 8 mg (Y=8%), UPLC purity: 92%, $[M+H]^+$=328.25 found.

^{1}H NMR (300 MHz, DMSO-d_6) δ 11.58 – 11.46 (m, 1H), 10.79 – 10.71 (m, 1H), 8.30 (s, 1H), 7.52 (d, J = 8.1 Hz, 1H), 7.43 – 7.35 (m, 3H), 7.32 – 7.24 (m, 3H), 7.23

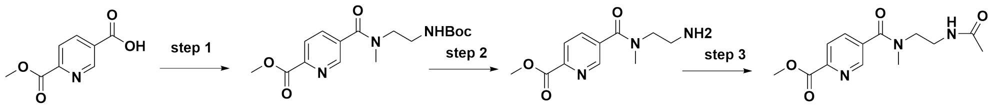

Fig. E6 Synthesis route for compound 24.

– 7.18 (m, 2H), 7.09 (t, J = 7.0 Hz, 1H), 6.59 – 6.56 (m, 1H), 6.23 – 6.18 (m, 1H), 4.13 (s, 2H).

2-benzyl-3-[(2E)-2-[(1H-1,2,3-triazol-4-yl)methylidene]hydrazin-1-yl]quinoxaline (22) 3 mg (Y=4%), UPLC purity: 92%, $[M+H]^+$=330.60 found.

Too small amount of material to perform good quality NMR.

2-benzyl-3-hydrazinylquinoxaline (23)

^{1}H NMR (300 MHz, DMSO-d_6) δ 7.52 – 7.42 (m, 2H), 7.40 – 7.35 (m, 2H), 7.32 – 7.24 (m, 3H), 7.22 – 7.18 (m, 1H), 7.08 – 7.01 (m, 1H), 4.10 (s, 2H), 2.10 (s, 1H), 2.01 (s, 2H).

E.6 Synthesis protocol for compound (24)

The synthesis route for compound 24 is shown in figure E6.

Step 1

To a solution of 2-Methyl hydrogen pyridine-2,5-dicarboxylate (200 mg, 1.0 eq) in anhydrous DMF (6.0 mL, 30 vol) at 0 °C DIPEA (8.0 eq) was added, then N-Boc-2-methylamino-ethylamine (1.0 eq) was added. After 15 min of stirring HATU (1.2eq) was added. The resulting mixture was stirred for 1.5 h at room temperature. The reaction mixture was quenched with water and extracted with EtOAc (x3). Combined organic layers were washed with brine, dried over anhydrous Na_2SO_4, filtered and concentrated. The crude material was purified by flash column chromatography (eluting with gradient DCM:acetone 1:0 → 0:1) to give 469 mg (Y=98%) of methyl 5-[(2-[(tert-butoxy)carbonyl]aminoethyl)(methyl)carbamoyl]pyridine-2-carboxylate with 78% UPLC purity. UPLC (ESI): exact mass for $C_{16}H_{23}N_3O_5$: 337.16; $[M+H]^+$=338.25 found.

Step 2

To solution of methyl 5-[(2-{[(tert-butoxy)carbonyl]amino}ethyl)(methyl)carbamoyl]pyridine-2-carboxylate (459 mg, 1.0 eq) in anhydrous DCM (18 mL, 40 vol) Hydrogen chloride, 4.0 M in dioxane (9.2 mL, 35.0 eq) was added dropwise at 0 °C. The resulting mixture was stirred for 2 h at 0 °C and the overnight at room temperature. The solvent was evaporated to obtain 408 mg (Y=quantitative) of methyl 5-[(2-aminoethyl)(methyl)carbamoyl]pyridine-2-carboxylate as a HCl salt. UPLC (ESI): exact mass for $C_{11}H_{15}N_3O_3$: 237.11; $[M+H]^+$=238.10 found.

Step 3

To a stirred solution of methyl 5-[(2-aminoethyl)(methyl)carbamoyl]pyridine-2-carboxylate (358 mg, 1.0 eq) in a mixture of DCM (2 mL, 6 vol) and 1.4-dioxane (10 mL, 10 vol) at room temperature, triethylamine (8.0 eq) and acetic anhydride (2.0 eq) were added. The resulting mixture was stirred for 30 min at room

temperature. Then the reaction mixture was quenched with water and extracted with DCM. Organic phase was dried over anhydrous Na_2SO_4, filtered and concentrated. The crude product was purified by flash column chromatography (eluting with gradient DCM:[DCM:MeOH = 9:1] 1:0 -¿ 1:1) to afford 168 mg (Y=66%) methyl 5-[(2-acetamidoethyl)(methyl)carbamoyl]pyridine-2-carboxylate with 95% UPLC purity. UPLC (ESI): exact mass for $C_{13}H_{17}N_{304}$: 279.12; $[M+H]^+$=280.70 found.

methyl 5-[(2-acetamidoethyl)(methyl)carbamoyl]pyridine-2-carboxylate (24) 12 mg (Y=32%), UPLC purity: 95%, $[M+H]^+$=280.70 found.

^{1}H NMR (300 MHz, DMSO-d_6) δ 8.77 – 8.61 (m, 1H), 8.13 – 7.97 (m, 2H), 7.97 – 7.79 (m, 1H), 3.90 (s, 3H), 3.52 (t, J = 5.9 Hz, 1H), 3.30 – 3.20 (m, 2H), 3.18 – 3.07 (m, 1H), 2.95 (d, J = 35.3 Hz, 3H), 1.76 (d, J = 31.0 Hz, 3H).

Appendix F Pharmacophore Models

All the MAO-A and MAO-B pharmacophore models determined by clustering the activity data are presented in Figures F7 and F8.

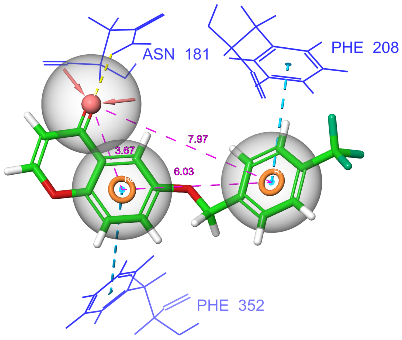

(a) Pharmacophore 1.

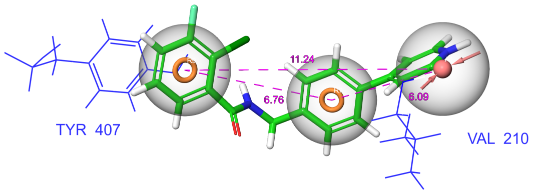

(b) Pharmacophore 2.

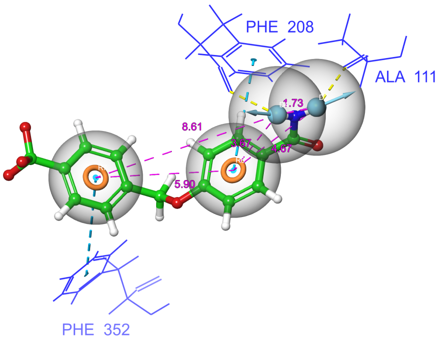

(c) Pharmacophore 3.

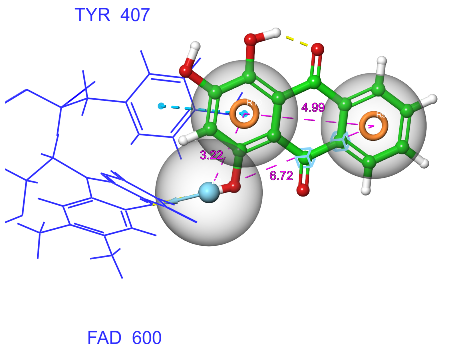

(d) Pharmacophore 4.

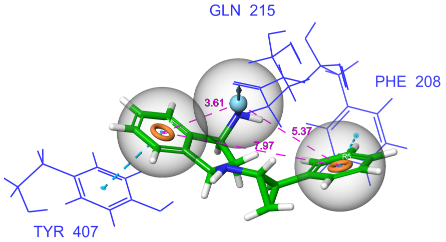

(e) Pharmacophore 5.

Fig. F7 MAO-A pharmacophore hypotheses. The orange rings represent aromatic fragments, cyan balls are hydrogen bond donors, and red balls are hydrogen bond acceptors.

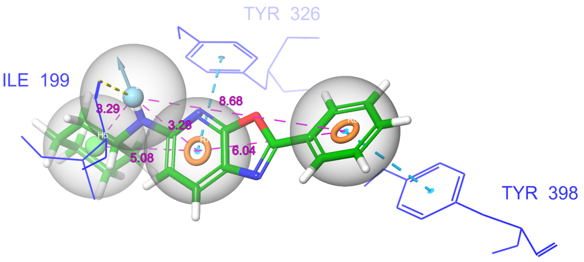

(a) Pharmacophore 1.

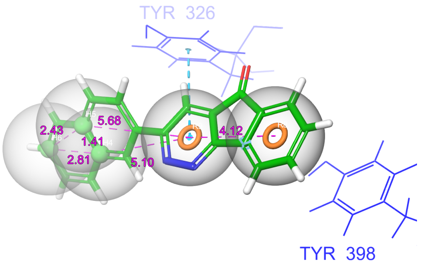

(b) Pharmacophore 2.

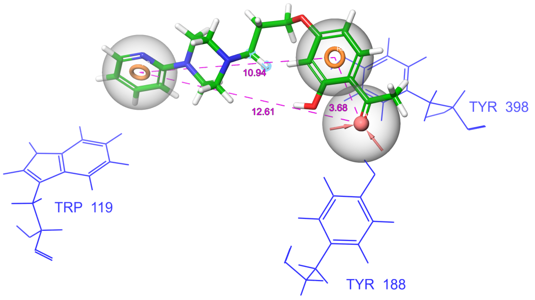

(c) Pharmacophore 3.

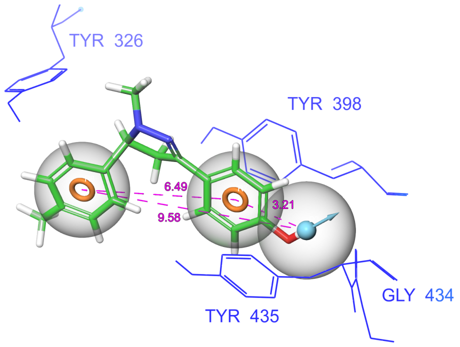

(d) Pharmacophore 4.

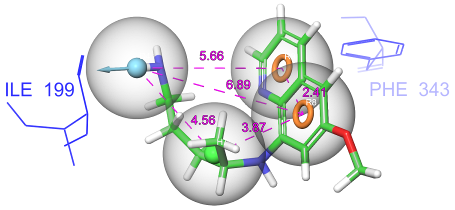

(e) Pharmacophore 5.

Fig. F8 MAO-B pharmacophore hypotheses. The orange rings represent aromatic fragments, cyan balls are hydrogen bond donors, red balls are hydrogen bond acceptors and lime balls are hydrophobic moieties.
